# Implementation outcomes from the Hypertension Treatment in Nigeria program: results from a type 2 hybrid interrupted time series trial

**DOI:** 10.1186/s13012-025-01472-1

**Published:** 2025-11-26

**Authors:** Jiancheng Ye, Lisa R. Hirschhorn, Abigail S. Baldridge, Erica L. Jamro, Ikechukwu A. Orji, Gabriel L. Shedul, Nanna R. Ripiye, Tunde M. Ojo, Helen Eze, Grace J. Shedul, Eugenia N. Ugwuneji, Rosemary C. B. Okoli, Boni M. Ale, Samuel Osagie, Olutobi A. Sanuade, Guhan Iyer, Namratha R. Kandula, Dike B. Ojji, Mark D. Huffman

**Affiliations:** 1 Northwestern University Feinberg School of Medicine, Chicago, IL USA; 2 Weill Cornell Medicine, Cornell University, New York, NY USA; 3 Washington University in St. Louis, St. Louis, MO USA; 4 Cardiovascular Research Unit, University of Abuja Teaching Hospital, Abuja, Nigeria; 5 University of Abuja, Abuja, Nigeria; 6 University of Nigeria, Nsukka, Nigeria; 7 Holo Global Health Research Institute, Nairobi, Kenya; 8 Department of Population Health Sciences, Division of Health System Innovation and Research, Spencer Fox Eccles School of Medicine, University of Utah, Salt Lake City, Utah USA; 9 The George Institute for Global Health, University of New South Wales, Sydney, Australia; 10 Department of Public Health, Federal of Ministry of Health and Social Welfare, Abuja, Nigeria; 11 School of Integrated Sciences, Sustainability, and Public Health, College of Health, Science, and Technology, University of Illinois Springfield, Illinois, USA

**Keywords:** Hypertension, Implementation outcome, Primary care, WHO HEARTS technical package, Quantitative analysis

## Abstract

**Background:**

The Hypertension Treatment in Nigeria Program was implemented across 60 primary healthcare centers (PHCs) in Nigeria to improve hypertension treatment and control using the World Health Organization’s HEARTS package. This study reports the program’s implementation outcomes.

**Methods:**

The Hypertension Treatment in Nigeria Program used a type 2 hybrid interrupted time series design, and data were collected from January 2020 to December 2023. The RE-AIM (Reach, Effectiveness, Adoption, Implementation, and Maintenance) framework guided the evaluation, focusing on key metrics such as patients’ and clinics’ characteristics, prescription rate of fixed dose combination (FDC) drugs, medication availability, and retention.

**Results:**

Among 21,922 patients recruited (mean [SD] age = 49 [12], 68.1% female) from 60 primary healthcare centers (78.3% rural). Prescription of FDC increased from 16.3 (95% CI: 4.8%—27.8%) to 65.2% (95% CI: 64.0%—66.3%). The program distributed 336,116 30-day medication supplies, and nearly all (95%) PHCs had at least one 30-day supply of any BP-lowering medication in stock after the drug revolving fund implemented. The patient retention rate at 6 months increased between the pre-implementation to implementation periods from 59.9% to 63.1%.

**Conclusions:**

The Hypertension Treatment in Nigeria Program successfully integrated hypertension services into Nigerian primary healthcare centers. Future efforts should focus on sustaining and scaling up the program’s success.

**Trial Registration:**

The trial has been registered at www.clinicaltrials.gov under NCT04158154.

**Supplementary Information:**

The online version contains supplementary material available at 10.1186/s13012-025-01472-1.

Contributions to the literature

- This study presents a detailed, Quantitative analysis of the Hypertension Treatment in Nigeria (HTN) Program’s implementation outcomes.
- This study highlights the need for well-defined and consistent measures of implementation outcomes of World Health Organization HEARTS (Healthy-lifestyle counselling; Evidence-based treatment protocols; Access to essential medicines and technology; Risk-based CVD management; Team-based care; Systems for monitoring) package using the RE-AIM ((Reach, Effectiveness, Adoption, Implementation, and Maintenance) framework.
- The findings provide policymakers and health system leaders with the evidence necessary to inform broader sustainment and dissemination efforts and to optimize the hypertension management program’s future scale-up.

## Introduction

Hypertension is a leading modifiable risk factor for cardiovascular disease (CVD) morbidity and mortality worldwide, significantly impacting both high-income and low- and middle-income countries (LMICs) [1, 2]. In response to the escalating global burden of CVD, the World Health Organization (WHO) developed HEARTS, a multi-level strategy package aimed at improving CVD screening, evaluation, and management with a focus on primary healthcare settings, particularly in resource-limited environments [3]. The HEARTS package, consisting of six modules, draws heavily from the successful Kaiser Permanente Northern California (KPNC) hypertension control program, which has been widely adopted and adapted [4]. Large-scale hypertension control initiatives in more than 30 countries have implemented HEARTS or KPNC-based models at both national and sub-national levels, demonstrating their adaptability and potential to improve blood pressure control [5–8].

In Nigeria, hypertension is a large and growing condition, affecting 29–38% of adults [9, 10]. To address this public health issue, we developed the Hypertension Treatment in Nigeria (HTN) Program, which leverages implementation science frameworks to contextualize, implement, and evaluate the multilevel implementation strategy package based on WHO HEARTS across 60 primary healthcare centers (PHCs) in the Federal Capital Territory, Nigeria [11]. Understanding the implementation process and corresponding implementation outcomes is critical to the evaluation of HEARTS, but such evaluations have been limited to date [12, 13].

In alignment with implementation science principles [14], this study aimed to address the gap in standardized implementation outcome reporting of HEARTS and understand the success in effectiveness. We sought to use well-defined measures to evaluate implementation outcomes and to ensure transparency and consistency in reporting across different settings [14]. The HTN Program protocol outlined and defined five key implementation outcomes at program, site, and patient levels based on the RE-AIM framework: reach, effectiveness, adoption, implementation, and maintenance [11]. The effectiveness results have been reported [15], and mixed methods evaluation will be reported separately.

The primary aim of this paper is to present a detailed, quantitative analysis of the HTN Program’s implementation outcomes. We also aim to highlight the need for well-defined and consistent measures of implementation outcomes of HEARTS using the RE-AIM framework [16]. This approach fosters comparability through transparent reporting and facilitates better understanding of factors contributing to the success or failure of implementation strategies [14]. Our findings will provide policymakers and health system leaders with the evidence necessary to inform broader sustainment and dissemination efforts and to optimize the program’s future scale-up.

## Methods

### Overview of the study design

The HTN Program used a type 2 hybrid interrupted time series design to simultaneously assess the effectiveness of a multi-level implementation package (i.e., adapted HEARTS) and its implementation within 60 PHCs in Nigeria. The HTN Program methods have been published and are briefly described here [11]. This design enabled us to generate novel information on both the package’s outcomes and its implementation, with the goal of informing subsequent regional- and national-scale up efforts [17]. The study design also allows for a comprehensive evaluation of the health system-, community-, site-, and individual-level characteristics that may influence effectiveness and implementation of the HTN Program [18].

### Timeline

The HTN Program followed a phased approach, starting with a formative period (April 2019 to December 2019), during which baseline quantitative service availability and qualitative assessments were conducted to inform the adaptation of the implementation package [19, 20]. The pre-implementation, control period (January 2020 to December 2020) included sequential registration and empanelment of hypertensive patients at participating PHCs. This was followed by the implementation period (January 2021 to December 2023), during which the full implementation package was introduced and evaluated. Analysis and dissemination of baseline results occurred following conclusion of the pre-implementation period [21]. Midway through the implementation period, we introduced the final patient-level component of the package, namely home blood pressure monitoring and health coaching led by community health extension workers (CHEWs) in a subset of 10 PHCs. This sub-study ran from June 2022 to December 2023 to evaluate effectiveness and implementation of patient-level home blood pressure monitoring and health coaching. The results of this sub-study will be reported separately.

### Site and participants

We conducted an initial assessment of all 243 public PHCs in the Federal Capital Territory to identify eligible sites for the study; Sixty PHCs were selected through a multi-stage random sampling process, stratified by patient load and geography and supplemented by priority PHCs identified by the National Primary Healthcare Development Agency [22]. All 60 invited sites agreed to participate. During both the pre-implementation and implementation periods, adults (≥ 18 years) presenting at participating sites with persistently elevated blood pressure (systolic ≥ 140 mm Hg or diastolic > 90 mm Hg, measured twice) or a history of hypertension were eligible for inclusion [23]. Data were collected using paper case report forms maintained by CHEWs and entered into a REDCap database by record information officers [24]. The forms captured sociodemographic, medical history, anthropometrics, blood pressure values, relevant laboratory studies (e.g., serum potassium, creatinine), medications, adverse events, counseling, and patient vital status.

### Implementation package

The adapted WHO HEARTS implementation package included a hypertension patient registry with empanelment of patients (health system level), monthly performance and quality reporting (health clinic level), simplified treatment guidelines (national policy level), encouragement and provision of FDC therapy (health system level), improved access to essential medicines and technology (health system level), team-based care and non-physician follow up (health worker level), and health coaching and home blood pressure monitoring (patient level), all of which have demonstrated improved population-level hypertension control in other settings [5, 25, 26]. The BP lowering medications have two protocols; Protocol 1 includes amlodipine, losartan, hydrochlorothiazide, amlodipine + losartan, amlodipine + losartan + hydrochlorothiazide; Protocol 2 replaced losartan with amiloride. We also developed a quarterly, supportive supervision program, community mobilization program, and drug revolving fund (a sustainable financing mechanism where patient payments for medications are reinvested to maintain drug supplies, reducing dependence on external funding while ensuring continuous availability) to improve site-level oversight, including longitudinal site-level data, community awareness about availability of hypertension services, and blood pressure medicine accessibility, respectively [27]. To prime the drug revolving fund and facilitate its use, blood pressure lowering medications were freely available from January 2021 to May 2022.

During the pre-implementation period, all sites were in the control phase of the program and a 2-day, in-person training was provided to a minimum of two CHEWs from each participating site, which was focused on measurement of blood pressure, diagnosis and management of hypertension, and record management [28]. Pre- and post-workshop tests were conducted to evaluate changes in knowledge and practice, including evaluation of accurate blood pressure measurement. Record officers at each site were also trained through an in-person half-day training on electronic data management, and pharmacy technicians were also trained on medication dispensation [29]. In January 2021, all sites transitioned to the implementation period of the program [30]. In November 2021, a two-day in-person training was provided to 30 CHEWs and 10 facility managers from 10 participating sites on how to carry out the home blood pressure and health coaching sub-study. The program’s primary outcomes, including differences in hypertension treatment and control, and blood pressure in the pre-implementation and implementation periods, have been reported [15].

### Implementation outcomes

Implementation outcomes focused on the domains outlined in the RE-AIM framework including at the program, site, and patient levels (Supplemental Table 1). We present the taxonomy and definitions, quantitative calculation methods, and data sources for the outcomes at multiple levels. Additionally, we included key results as illustrative examples in the RESULTS section. We used quantitative analysis based on the RE-AIM framework to triangulate routinely collected data to evaluate available quantifiable variables of the implementation package [16]. Each construct was mapped to specific metrics, such as patients’ and clinics’ characteristics, prescription of FDC drugs, medication availability, and retention rates, which were derived from a combination of routinely collected data, surveys, and administrative records. Walk-in treatment is defined as the proportion of eligible patients treated at the start of a patient’s baseline visit, while walk-out treatment refers to proportion of eligible patients treated at the conclusion of each visit to differentiate previous versus new prescriptions. The retention, or follow-up, rate was calculated by dividing the number of patients who had a follow-up visit within a prespecified number of days (i.e., 6 months) after their registration visit by the total number of registered patients with hypertension; we excluded patients who were ineligible for follow-up within 6 months due to the Program’s timeframe (i.e., registered after June 2023).

### Statistical analyses

We utilized both statistical summaries and multi-level analyses to quantify implementation outcomes, ensuring that variability across sites, subgroups, and time phases was captured. Descriptive data are reported as means (standard deviation) or medians (interquartile ranges, IQR) for continuous variables if data were skewed, and as proportions (95% CI) for categorical variables. Categorical variables are described as frequencies and percentages. Comparisons were made using t-tests, Mann–Whitney U tests, and Chi-square tests, based on the outcomes. Interrupted time series analyses were performed to evaluate linear and nonlinear trends in retention rates before and after the implementation period of the HTN Program. Further details of more complex implementation outcomes are provided in supplemental Tables and Figures. Data were analyzed overall and by key subgroups, including by sex, PHC, and area council. Missing data were identified through bi-weekly data quality reports and resolved with the responsible site [11] and the final missing rates for all the outcomes were less than 5% [15]. All analyses were completed using a complete case approach. The intraclass correlation coefficient (ICC) was calculated at the PHC level to assess clustering of retention rates within facilities. SAS version 9.4 (SAS, Cary, NC, USA) and R version 4.0.5 (R Foundation, Vienna, Austria) were used for statistical analyses. A two-sided *p*-value < 0.05 was used to define statistical significance, and no adjustments were made for multiple comparisons.

## Results

### Reach

Table 1 presents the distribution and characteristics of 60 PHCs based on patient numbers, visit counts, rural representation, and characteristics of patients across six area councils. A total of 21,922 patients were recorded across the PHCs, with the Abuja Municipal Area Council contributing the highest percentage at 31.5%. Supplemental Fig. 2 shows the participant enrollment and inclusion. The total number of visits was 142,493. Out of 60 PHCs, 47 (78.3%) were in rural areas. The average age of all patients was 49.5 years (SD = 12.5); females constituted a significant majority of the population in all areas.

**Table 1:** PHCs’ size, diversity of PHCs, and characteristics of sociodemographic by areas

PHCs (*N* = 60)	Patients, *N* (%)	Visits, *N* (%)	PHCs in rural areas, *N* (%)	Age, mean (SD)	Female, *N* (%)	No education, *N* (%)
Abaji (*N* = 8)	3227 (15.0%)	29,144 (20.5%)	6 (75%)	53.4 (12.5)	2212 (67.4%)	1545 (47.9%)
Abuja Municipal Area Council (*N* = 15)	6861 (31.5%)	31,356 (22.0%)	12 (80%)	47.0 (12.7)	4966 (71.9%)	1148 (16.8%)
Bwari (*N* = 8)	3243 (15.0%)	19,544 (13.7%)	5 (62.5%)	48.8 (11.8)	2219 (67.3%)	471 (14.6%)
Gwagwalada (*N* = 11)	4201 (19.4%)	33,479 (23.5%)	7 (63.6%)	49.8 (11.8)	2894 (68.0%)	1261 (30.1%)
Kuje (*N* = 8)	1968 (9.2%)	16,320 (11.5%)	7 (87.5%)	51.5 (12.1)	1325 (65.5%)	559 (28.4%)
Kwali (*N* = 10)	2113 (9.9%)	12,650 (8.9%)	10 (100%)	49.9 (12.6)	1326 (61.1%)	827 (39.3%)
Total (*N* = 60)	21,922 (100%)	142,493 (100%)	47 (78.3%)	49.5 (12.5)	14942 (68.2%)	5811 (27%)

*Abbreviation*: *PHC* primary healthcare center

Supplemental Table 2 presents the diversity of healthcare staff across various area councils categorized into permanent and per-diem staff, as well as community volunteers at the baseline supervision visit. The total number of healthcare staff reported was 1,183, with significant variation across the different area councils. A total of 503 permanent staff members were documented. Notably, CHEWs represented the largest proportion (47%) of permanent staff. The presence of medical doctors (1%), pharmacy staff (1%), and paramedical professionals (1%) remained low across all areas.

Supplemental Table 3 presents the sociodemographic and clinical characteristics of patients between the pre-implementation and implementation periods. One-quarter (25.1%) of patients were enrolled during the pre-implementation period, and three-quarter (74.9%) were enrolled during the implementation period. Female patients comprised the majority of participants (68.2%), with a small but statistically significant increase in their proportion during the implementation period (66.6% to 68.7%, *P* = 0.003). Baseline systolic and diastolic BP values were slightly higher during implementation. Walk-out treatment significantly increased during the implementation period, rising from 91.4% in pre-implementation period to 96.9% (*P* < 0.0001).

### Effectiveness

The program’s effectiveness outcomes have been reported separately [15]. By the end of the study period, the treatment rate had increased from 86.2% (95% CI: 83.5%, 88.8%) to 96.4% (95% CI: 96.1%, 96.7%), and the control rate from 21.7% (95% CI: 18.5%, 24.8%) to 55.9% (95% CI: 55.1%, 56.7%).

### Adoption

Table 2 presents the prescription of fixed dose combination (FDC) therapy across various PHCs in different areas. Across all 60 primary healthcare centers (PHCs), prescription of FDC medications increased significantly from 16.3% (95% CI: 4.8%, 27.8%) in the pre-implementation period to 65.2% (95% CI: 64.0%, 66.3%) in the implementation period (*P* < 0.001).

**Table 2:** Fixed dose combination therapy rate by areas and implementation period

PHCs (*N* = 60)	Pre-implementation, % (95% CI)	Implementation, % (95% CI)	*P* Value
Abaji (*N* = 8)	20.8 (6.0, 36.4)	83.1 (79.9, 86.9)	<.0001
Abuja Municipal Area Council (*N* = 15)	10.2 (3.0, 17.9)	41.0 (39.4, 42.7)	<.0001
Bwari (*N* = 8)	25.0 (7.2, 43.6)	95.9 (86.5, 93.5)	<.0001
Gwagwalada (*N* = 11)	18.1 (5.2, 31.8)	72.4 (68.8, 75.9)	<.0001
Kuje (*N* = 8)	19.4 (5.4, 34.7)	77.5 (72.4, 82.7)	0.03
Kwali (*N* = 10)	18.2 (5.1, 32.6)	72.7 (68.5, 77.6)	<.0001
Total (*N* = 60)	16.3 (4.8, 27.8)	65.2 (64.0, 66.3)	0.0004

The *P* values compare the fixed dose combination therapy rate at the pre-implementation and implementation periods

*Abbreviation:* *CI* confident interval, *PHC* primary healthcare center

### Implementation

Supplemental Fig. 1a and Supplemental 1b outline the availability of BP-lowering medications across area councils, comparing periods when drugs were freely available and when the drug revolving fund was implemented. Across all regions and years, there was an overall major increase in the availability of BP-lowering medications (*P* value for trend < 0.0001). A total 336,116 doses of 30-day medications were distributed by the program.

### Maintenance

Table 3 presents the proportion of PHCs that have at least one 30-day supply of any BP-lowering medication in stock based on the treatment protocols. In 2020, the proportion of PHCs with at least one 30-day supply of BP-lowering drugs was relatively low, with 27% prior to the availability of free medications. Availability of medications in both protocols improved throughout the study period, reaching 96% by the end of the study. There were 73.3% of PHCs that maintained hypertension control above baseline rate (13.1%) at 6 months, which increased to 88.3% at 12 months and 96.7% at 24 months.

**Table 3:** Proportion of PHCs that have at least 1 30-day dose of any BP-lowering drug in stock

	*Baseline*			*Drugs freely available*						*Drug revolving fund implemented*						
	2020			2021				2022				2023				
Drugs	Q2ᵈ	Q3	Q4ᶠ	Q1ᵉ	Q2	Q3	Q4	Q1ᵃ	Q2ᵉ	Q3ᵇ	Q4	Q1	Q2ᶜ	Q3	Q4ᶜ	*P* Value
**Protocol**^†^	27%	28%	20%	77%	82%	78%	92%	93%	89%	84%	93%	98%	98%	100%	96%	< 0.001

Primary healthcare center inventories missing: ᵃ*n*=1; ᵇ*n*=2; ᶜ*n*=4; ᵈ*n*=5; ᵉ*n*=7; ᶠ*n*=10

^†^Including Protocol 1 and 2. Protocol 1: Blood pressure lowering medications including: amlodipine, losartan, hydrochlorothiazide, amlodipine + losartan, amlodipine + losartan + hydrochlorothiazide; Protocol 2 replaces losartan with amiloride. The *P* value compare the proportion of primary healthcare centers that have at least 1 30-day dose of any blood pressure-lowering drug in stock at the pre-implementation period and implementation period

**Fig. 1:**
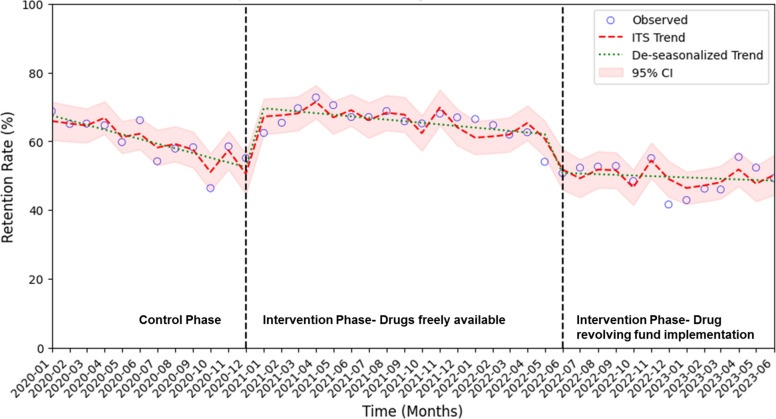
Interrupted time series 6-month rolling average retention rate by implementation period. Monthly point estimates are based on the observed retention rate, determined from the date of the first (i.e., registration) clinic visit. Circles indicate the mean 6-month retention rate in each month; shaded area, 95% CIs; dashed line, predicted 6-month retention rate trend based on the seasonally adjusted regression model; dotted line, deseasonalized trend. The implementation period was divided into two phases: (1) the free drug distribution phase, and (2) the Drug Revolving Fund (DRF) implementation phase. Abbreviation: CI, confidence interval; ITS, interrupted time series

Figure 1 shows the 6-month retention rate by area councils and implementation period. The implementation period was divided into two stages: (1) the free drug distribution phase, and (2) the Drug Revolving Fund (DRF) implementation phase. The nonlinear trend revealed a potential pattern of seasonality, with higher retention rates after baseline visits as well as around April and December. The slope of the retention rate increased during the implementation period compared with the pre-implementation period, especially during the DRF implementation phase. Supplemental Table 4 demonstrates the retention rates across six area councils at intervals of 3, 6, 12, and 24 months, during the pre-implementation and implementation periods. At 6 months, the overall retention rate rose from 59.9% to 63.1% (ICC = 0.29). Retention rates showed a stepwise increase with longer time horizon: at 24 months, there was a slight improvement from 70.6% to 71.8%. Abuja Municipal Area Council and Bwari consistently demonstrated statistically significant improvements in retention during the implementation period.

## Discussion

### Summary of Results

This evaluation provides a comprehensive analysis of the HTN Program’s implementation outcomes in 60 PHCs across the Federal Capital Territory of Nigeria. The program achieved notable reach across diverse PHCs, with 78.3% located in rural areas, highlighting its emphasis on improving access to underserved populations. The inclusion of large population of 21,922 patients, the majority of whom were female (68.2%) also points to the wide reach of this program. Adoption of FDC therapy was a key success of the program, increasing from 16.3% at baseline to 65.2% by study end, which contributed increases in hypertension control. The implementation of the drug revolving fund substantially improved medication availability, with large increases in the proportion of PHCs that maintained a 30-day supply by the study’s end, even after the free supply of medication ended.

### Synthesis of results

Our motivation to conduct a quantitative analysis of implementation outcomes stems from the need to assess the reach, effectiveness, adoption, fidelity, and maintenance of the HTN Program to inform sustainability and scale-up. Quantifying these outcomes allows us to systematically examine the factors that contribute to the success or challenges of implementing the intervention in different healthcare settings.

The evaluation of the HTN Program reveals several key trends. One of the program’s significant achievements is the broad geographic coverage and inclusion of predominantly rural PHCs, demonstrating the program’s ability to reach historically underserved populations. The substantial improvements in FDC therapy rates suggest that the simultaneous use of standardized treatment protocols, training and supportive supervision, and consistent FDC supply through the drug revolving fund implementation, effectively addressed barriers to hypertension management. The variability in medication availability across area councils highlights the ongoing need to refine strategies for ensuring consistent access to essential drugs, particularly in rural areas.

Patient retention patterns showed encouraging improvement throughout the program implementation. During the pre-implementation period, retention rates averaged 59.9% at six months, with notable seasonal fluctuations that peaked around April and December. Following program implementation, retention improved to 63.1% at six months, maintaining similar seasonal patterns but at consistently higher levels. The transition to the drug revolving fund phase demonstrated sustainability, as retention rates remained stable despite patients beginning to pay for medications, supporting the program’s goal of sustained engagement with primary healthcare services.

The adoption of FDC therapy demonstrated major implementation changes. Prior to program implementation, only 16.3% of patients received FDC medications, reflecting limited availability and prescribing practices focused on single-agent therapies. During the implementation period, FDC prescribing increased to 65.2%, representing a four-fold improvement compared to the pre-implementation period. This shift toward simplified medication regimens aligns with WHO HEARTS recommendations and international evidence showing that FDC therapy reduces pill burden and improves medication adherence compared to multiple separate medications [31].

Medication availability patterns demonstrated the critical role of supply chain improvements in supporting clinical care. The establishment of drug revolving funds addressed one of the most persistent barriers to hypertension management in resource-limited settings. Nearly all participating healthcare centers maintained adequate medication supplies by the study’s conclusion, ensuring that improvements in prescribing practices could translate into patient accessibility of essential medications.

The retention rates improved significantly over time, with a 24-month retention rate of 71.8% among patients across all regions [32]. Retention rates appeared to be higher not only following baseline visits but also during certain months, particularly around April and December. The slight decrease of the retention rate at the end of the study may due to a few reasons: 1) as the patient population increased by time, clinics may have had less capacity to conduct routine follow-up of empaneled patients, such as phone calls; or 2) the proposed implementation package, including monthly follow up, might be perceived by patients as too burdensome [32]. Sustained retention is critical for long-term hypertension management, and future initiatives should explore strategies such as longer term medication prescriptions, community outreach programs, or identifying high-risk or high-priority participants who may require intensive follow-up and tailor resources accordingly [33]. Some HEARTS Programs offer extended (60- or 90-day) medication refills, which permits less frequent visits to the primary care facility and promotes adherence [34]. However, the out-of-pockets cost for one month of BP lowering medications was considered expensive for many patients in this program, making longer-term prescriptions unaffordable without expansion of community insurance benefits. The estimated medication cost during the free-drug phase was approximately USD $3.20 per patient-month, covering medication-related training, supervision, and monitoring. During the drug revolving fund phase, patient co-payments averaged USD $1.60 per patient-month, with revenues used to sustain stock replenishment [35]. These costs are similar to or lower than those reported by other similar programs, such as the Nigeria Hypertension Control Initiative, supporting the potential feasibility and affordability of scaling the program [34].

### Comparison with previous research

In the context of dissemination and implementation science, clear and consistent reporting of implementation outcomes is critical for evaluating the success and sustainability of implementation strategies. The dissemination and implementation science community emphasizes the need for rigorous and systematic definition and measurement of outcomes to ensure clarity and comparability across studies [14, 36]. These recommendations provide a framework for strengthening implementation science by addressing issues like specifying the operational definitions of outcomes, using well-defined measures, and reporting both process and outcome indicators [37].

The HTN Program’s implementation of the adapted WHO HEARTS technical package aligns with global efforts to improve cardiovascular disease prevention and control in primary healthcare settings [34]. The HEARTS package proposed a short list of core indicators: the number of hypertensive patients enrolled from the catchment area, proportion of enrolled patients with BP controlled, and proportion of patients not retained in care [34]. By the end of 2022, 32 LMICs were actively participating in the Global Hearts Program, implementing one or more components of the WHO HEARTS technical package [34]. Spanning a catchment area that encompasses 144.0 million individuals living with hypertension, the program enrolled a total of 12.2 million hypertensive patients across 165,107 primary health care facilities. Additionally, over 399,000 healthcare workers were trained to implement the HEARTS package. At the international level, median hypertension control was 48%, ranging from 5 to 86%. In Nigeria, HEARTS implementation in Kano and Ogun states through the Nigeria Hypertension Control Initiative over the same period demonstrated an endline hypertension control proportion of 23.1% among 36, 345 patients [34].

The median rate of missed scheduled visits within the previous three months was 35%, with a range of 1% to 79% [34]. Retention is a common and pervasive, yet understudied, issue in hypertension control programs worldwide, as reflected in missed visit rates. The HEARTS initiative in Guatemala reported 3-month retention rate as 36%, and the treatment was only 22.3% [38]. Even countries with moderate success in BP control, like Vietnam (30.5%), faced challenges with retention, as evidenced by a 52% missed visit rate within 3 months [39]. In the HTN Program, the 3-month retention rate was 57.2% (missed visit rate: 42.8%), and 6-month retention rate was 63.1% (missed visit rate: 36.9%), compared with a missed visit rate of 56.2% in the Nigeria Hypertension Control Initiative [34]. These findings highlight the need for targeted strategies to reduce patient attrition, including improved follow-up systems, enhanced patient education, and addressing barriers to consistent care [33].

The HEARTS Program has been implemented across Sub-Saharan Africa, South Asia, East and Southeast Asia, and Latin America and the Caribbean. India and Thailand initiatives demonstrated large-scale enrollment, with over 4 million and 3 million hypertensive patients enrolled, respectively, reflecting the vast reach of their programs [40]. In contrast, smaller-scale programs such as Sri Lanka enrolled 5,306 patients [41]. The HTN Program enrolled 21,922 patients, which was the most effective and second largest HEARTS Program in Africa.

The proportion of enrolled patients with BP controlled varied widely in other HEART-implemented programs. Ethiopia’s program [42] achieved relatively high control rates (46%), whereas countries like China (4.9%) struggled to achieve meaningful control among their enrolled populations [43]. In Bangladesh, a quasi-experimental trial assessing the WHO HEARTS hypertension control package demonstrated a significant 12.3% increase in blood pressure control rates [44]. These differences underscore the influence of regional challenges, such as medication availability, health system infrastructure, and local contexts. The India Hypertension Control Initiative successfully decentralized care to primary health care centers and community-based Health and Wellness Centers. These facilities achieved hypertension control rates of 46% and 42%, respectively, by March 2020, outperforming the 35% control rate observed in more centralized district hospitals [45].

A review of hypertension control interventions in LMICs found that multi-component interventions, like the WHO HEARTS package, were more effective than single-component interventions [34]. Our results supports this finding, as the HTN Program’s multi-level package led to improvements across various outcomes. Compared to other package implementations, our study’s focus on FDC therapy is noteworthy because this approach can help promote programmatic success. A major barrier to improving hypertension control in many regions implementing the WHO HEARTS package is the scarcity of medications. Best practices to address this barrier include selecting locally available drugs, maintaining buffer stocks, proactively restocking facilities, and using effective stock management to prevent shortages. Long-term solutions involve using FDCs, streamlining procured medications to one per class, and establishing efficient procurement systems with benchmark pricing as has been done in HEARTS in the Americas [46, 47].

Overall, the HTN Program’s success in improving hypertension treatment and control, medication availability, and retention rates demonstrates the adaptability and potential impact of the HEARTS package in resource-limited healthcare environments. These comparisons emphasize the importance of context-specific adaptations and need for ongoing research to optimize the implementation of such packages in diverse settings. By incorporating implementation outcomes, the study generated valuable insights into the factors that influenced the results and identified key lessons for enhancing future interventions.

From this evaluation, we identified several specific lessons for future implementation of HEARTS-based programs. First, sustainable medication accessibility through mechanisms such as drug revolving funds is essential for program continuity [48]. Second, standardized treatment protocols and promotion of fixed-dose combinations may accelerate treatment adoption and improve hypertension control. Third, leveraging task-sharing with community health extension workers supports scalability and rural coverage [27]. Finally, sustained patient retention remains challenging; context-based strategies addressing local transportation, communication infrastructure, and patient demographics, as well as community-based follow-up through differentiated service delivery models should be tested [33].

### Strengths

This study has several strengths that contribute to its value. First, the use of the RE-AIM framework allowed for a structured and comprehensive evaluation of the program’s outcomes. By systematically assessing Reach, Effectiveness, Adoption, Implementation, and Maintenance, the study provides a holistic view of the program’s performance, ensuring that its impact is captured across multiple domains. This is among the first studies to provide a comprehensive taxonomy of HEARTS implementation in routine primary care (detailed in Supplemental Table 1) and conduct quantitative analysis for implementation outcomes based on the RE-AIM framework in routine primary care. This approach also facilitates comparisons with HEARTS Programs globally, enhancing the broader applicability of the findings. Second, the large sample size of 60 PHCs and geographic diversity of the study areas significantly increase the generalizability and representativeness of the results. The inclusion of both urban and rural PHCs provides a nuanced understanding of the program’s performance across varied contexts. This diversity allows for insights into how the implementation package can be adapted to different levels of infrastructure, resource availability, and population characteristics, making the findings relevant not only to Nigeria but also to other low-resource settings with similar healthcare challenges in Africa and beyond. Third, the study’s interrupted time series design strengthens the causal inferences that can be drawn about the program’s effectiveness and implementation. This design allowed for the tracking of changes over time, distinguishing the implementation package’s impact from underlying trends, and providing a robust understanding of the temporal effects of the program and its sustainment.

### Limitations

This study also has several limitations. First, data were collected from PHCs in the Federal Capital Territory of Nigeria, which may limit the generalizability of the findings to other regions or countries with different healthcare infrastructure, resources, and patient populations. The site selection may have led to an overestimation of program effectiveness compared to a scenario of mandatory implementation across all facilities, as participating sites generally had stronger baseline capacity, better staffing, and a demonstrated willingness to engage. Nonetheless, the predominantly rural sample (78.3%) and low baseline hypertension control rate (13.1%) indicate that significant implementation challenges persisted even within these relatively prepared sites. The findings are most applicable to similar primary care settings in Nigeria and other LMICs that possess basic health system infrastructure and trained community health workers. However, they may not reflect outcomes in facilities that lack minimum readiness criteria, particularly if implementation were mandated without adequate support. Importantly, the HTN Program was implemented across both urban and rural settings, encompassing PHCs with varied characteristics, such as differences in staffing levels and resource allocation. This diversity in settings may mitigate the risk of bias in implementation outcomes. Second, this study primarily focused on implementation outcomes using the RE-AIM framework, which, while comprehensive, may not capture the full spectrum of factors influencing program implementation. For example, contextual elements such as local health policies, social and structural determinants of health, and cultural factors were not systematically measured but may have played a role in the program’ s implementation and outcomes. However, our team has used qualitative methods and system-based modeling approaches in evaluations that may provide a more holistic understanding of these contextual influences and will be reported separately [33]. Future studies could also incorporate multilevel or stratified ITS models to explore effect heterogeneity. Third, because comparable patient-level data were not systematically collected from non-participating PHCs during the study period, which precluded the use of more robust comparative analysis; a controlled ITS or difference-in-differences design would provide stronger causal inference. Fourth, hypertension control rates were calculated among patients with documented follow-up visits within the specified timeframe, which may overestimate population-level control. Patients lost to follow-up may have worse blood pressure control than those retained in care. This represents a persistent challenge for large-scale, longitudinal hypertension programs even with systematic empanelment and tracking systems [49]. The retention rates we observed are comparable to other HEARTS programs but highlight that sustained engagement remains a major hurdle requiring continued innovation in service delivery models. Future hypertension programs should implement strategies to minimize loss to follow-up and report retention results.

Overall, this evaluation demonstrates that large-scale implementation of a hypertension control program based on the WHO HEARTS technical package is feasible and effective in Nigeria, a low-resource setting, and achieved most of the targeted implementation outcomes based on the RE-AIM framework including at the program, site, and patient levels. Future research should focus on sustainment and scale-up of implementation package [50, 51].

## Conclusion

This study provides a comprehensive evaluation of the implementation outcomes of the HTN Program, aimed at improving hypertension management in PHCs across the Federal Capital Territory of Nigeria. Using the RE-AIM framework, we systematically quantified the program’s reach, effectiveness, adoption, implementation, and maintenance, offering valuable insights into its performance in a low-resource setting. Overall, the HTN Program shows that large-scale hypertension control can be implemented and effective in primary care settings in Nigeria, particularly when leveraging community health workers and improving medication supply chains. Lessons learned from this evaluation can inform sustainment and scale-up of this program in Nigeria, as well as the design and scaling of HEARTS in other LMICs, contributing to global efforts to control hypertension and reduce the burden of cardiovascular diseases.

## Supplementary Information

Supplementary Material 1.

## Data Availability

The datasets and code generated and/or analyzed during the current study will be available in the NHLBI BioLINCC repository, https://biolincc.nhlbi.nih.gov/home/ after completion of the study.

## Abbreviations

CHEW: Community health extension worker
CLD: Causal loop diagram
CVD: Cardiovascular disease
HEARTS: Healthy-lifestyle counselling; Evidence-based treatment protocols; Access to essential medicines and technology; Risk-based CVD management; Team-based care; Systems for monitoring
HTN: Hypertension Treatment in Nigeria Program
ICC: Intra-class correlation coefficients
LMICs: Low- and middle-income countries
PHC: Primary healthcare center
RE-AIM: Reach, Effectiveness, Adoption, Implementation, and Maintenance
REDCap: Research Electronic Data Capture
WHO: World Health Organization
