# Supplementary material for: Implementation outcomes from the Hypertension Treatment in Nigeria program: results from a type 2 hybrid interrupted time series trial
Source: Implement Sci. 2025 Nov 26;21:1. doi: 10.1186/s13012-025-01472-1 (PMC12763998; doi:10.1186/s13012-025-01472-1)
Supplement: Supplementary file 1 — Supplementary Material 1. [file 13012_2025_1472_MOESM1_ESM.docx]

**Supplemental Tables/Figures**

**Supplemental Table 1.** Definition and method of calculation for the quantitative implementation outcomes of HTN Program**.**

| **RE-AIM Domain** | **Level** | **Outcome** | **Method of calculation** | **Results^*^** | **Data source** |
| --- | --- | --- | --- | --- | --- |
| **REACH:**  **Absolute number, proportion, and representativeness of sites and individuals who participate in the HTN Program** | Program | Number of participating PHCs/total number of selected PHCs in the FCT | A = Number of participating PHCs  B = Total number of selected PHCs in the FCT  Calculation: A ÷ B | 24.7% (60/243) | Registry data |
|  | Program | Number of participating PHCs/total number of PHCs that were reached out in the FCT | A = Number of participating PHCs  B = Total number of PHCs that were reached out in the FCT  Calculation: A ÷ B | 100% (60/60) | Registry data |
|  | Center | Diversity of participating PHCs and staff in terms of size, ward, baseline staffing levels | PHCs’ size = Number of patients; Number of visits  Diversity of PHCs in terms of ward = Number of PHCs in each area council  Diversity of staff = Number of staff in each area council at the baseline supervision visit | Supplemental Table 2 | Registry data, supervision data |
|  | Individual | Number of adult patients with BPs measured / total number of adult patients within participating PHCs within the given dates | Total number of adult patient visits to the health facility within the given dates documented at the first and last supportive supervision visits, N  Of these adult patients, how many had their BP checked during their visit? N (%)  Of the patients who had their BP checked, how many had high BP? Median N (%) | Supervision paper [27] | Supervision data |
|  | Individual | Differences in sociodemographic (e.g., age, sex, education, geography) characteristics between registered patients and individuals in the clinic catchment areas based on concurrently collected or community-based survey data | Differences in sociodemographic (age, sex, no education, rural) characteristics of registered patients. | Table 1 | Registry data |
|  | Individual | Diversity of registered patients receiving care at participating PHCs for HTN diagnosis and management by age, sex, ward, and education | Patients’ sociodemographics, clinical characteristics, and blood pressure overall and by sex. | Supplemental Table 3 | Registry data |
| **EFFECTIVENESS: The impact of the HTN Program on treatment and control rates** | Program | Treatment and control rates within the overall system of participating PHCs for the entire study period, defined by 6-month rolling average  The primary effectiveness outcome will be to compare the slope before and after the intervention period | 6-month rolling average treatment rate for all the PHCs  6-month rolling average control rate for all the PHCs | Primary outcomes paper [15] | Registry data |
|  | Program | Mean SBP and DBP within the overall system of participating PHCs for the entire study period, defined by 6-month rolling average and based on last visit | 6-month rolling average SBP for all PHCs  6-month rolling average DBP for all PHCs  Last visit’s SBP for all PHCs  Last visit’s DBP for all PHCs |  | Registry data |
|  | Center | Treatment and control rates across  participating PHCs for the entire study period, defined by 6-month rolling average | 6-month rolling average treatment rate  6-month rolling average control rate |  | Registry data |
|  | Center | Mean SBP and DBP across participating PHCs for the entire study period, defined by 6-month rolling average and based on last visit | 6-month rolling average SBP  6-month rolling average DBP  Last visit’s SBP  Last visit’s DBP |  | Registry data |
| **ADOPTION: Absolute number, proportion, and representativeness of sites who are willing to initiate the HTN Program** | Program | Use of HTN registry within individual participating PHCs by 3-months of site initiation | A = Number of PHCs that used HTN registry by 3-months  B = Total number of PHCs in HTN Program  Calculation: A ÷ B | 100% (60/60) | Supervision data |
|  | Program | Percentage of patients treated with fixed dose combination therapies in the last 3-months | A = Number of patients in HTN Program that were treated with fixed dose combination therapies in the last 3 months.  B = Total number of patients in HTN Program  Calculation: A ÷ B | Primary outcomes paper [15] | Registry data |
| **IMPLEMENTATION: Fidelity to the HTN Program protocol, including consistency of delivery as intended. Time and cost of the intervention, and use of the intervention strategies** | Program | Proportion of selected PHCs who participated in baseline hypertension training | A = Number of PHCs who participated in baseline hypertension training  B = Total number of selected PHCs  Calculation: A ÷ B | Supervision paper [27] | Supervision data |
|  | Program | Proportion of selected PHCs who participated in site initiation training | A = Number of PHCs who participated in site initiation training  B = Total number of selected PHCs  Calculation: A ÷ B |  | Supervision data |
|  | Program | Proportion of selected PHCs who received at least one supportive supervision visit in the past 7-months | A = Number of PHCs who received at least one supportive  supervision visit in the past 7-months  B = Total number of selected PHCs  Calculation: A ÷ B |  | Supervision data |
|  | Program | Proportion of selected PHCs who received an audit and feedback report within the past 3-months | A = Number of PHCs who received at least an audit and feedback  report within the past 3-months  B = Total number of selected PHCs  Calculation: A ÷ B |  | Supervision data |
|  | Program | Percentage of PHCs with a working blood pressure monitor at the site on the day of assessment | A = Number of PHCs with a working blood pressure monitor at the site on the day of assessment (time point: last supervision visit)  B = Total number of selected PHCs  Calculation: A ÷ B |  | Supervision data |
|  | Program | Percentage of PHCs with blood pressure medicines available on the day of assessment | A = Number of PHCs with blood pressure medicines available on the day of assessment (time point: last supervision visit)  B = Total number of selected PHCs  Calculation: A ÷ B |  | Supervision data |
|  | Program | Percentage of patients with step up indicated who received step up treatment in the last 6-months | A = Number of visits that patients who received stepped up treatment in their first four visits in the last 6-months  B = Total number of visits that patients need to receive stepped up treatment  Calculation: A ÷ B | Visit 1=37.7% (95%CI: 36.85%-38.5%)  Visit 2=7.1% (95%CI: 6.5%-7.7%)  Visit 3=1.3% (95%CI: 1.0%-1.6%)  Visit 4=0.4% (95%CI: 0.2%-0.6%) | Registry data |
|  | Center | Number and proportion of adult patients with hypertension who are registered/total number of adult patients with elevated blood pressure within participating PHCs within the past 3 working days | A = Total number of adult patients who had high BP visits to the health facility within the given dates  B = Total number of patients visits to the health facility within the given dates  Calculation: A ÷ B, 3-monthly rolling average rate | Supervision paper [27] | Supervision data |
|  | Center | Monthly proportion of registered patients with appropriate stepped care/total number of registered patients | A = Number of patients who need step up treatment and received it  B = Total number of patients who are registered per month  Calculation: A ÷ B, 3-monthly rolling average rate | Primary outcomes paper [15] | Registry data |
|  | Center | Monthly proportion of registered patients treated with fixed dose combination therapy/total number of patients on treatment | A = Number of patients who are treated with fixed dose combination therapy  B = Total number of patients who are treated per month  Calculation: A ÷ B | Table 2 | Registry data |
| **Maintenance:**  **The extent to which the HTN Program becomes institutionalized or part of the routine organizational practice** | Program | Proportion of participating PHCs who maintain treatment rates above baseline rates at 6, 12, and 24 months | A = Number of PHCs who maintain treatment rates above baseline rates (58.3%) at 6, 12, and 24 months  B = Total number of selected PHCs  Calculation: A ÷ B | 6 Month: 95.0% (57/60)  12 Month: 96.7% (58/60)  24 Month: 98.3% (59/60) | Registry data |
|  | Program | Proportion of participating PHCs who maintain control rates above baseline rates at 6, 12, and 24 months | A = Number of PHCs who maintain control rates above baseline rates (13.1%) at 6, 12, and 24 months  B = Total number of selected PHCs  Calculation: A ÷ B | 6 Month: 73.3% (44/60)  12 Month: 88.3% (53/60)  24 Month: 96.7% (58/60) | Registry data |
|  | Program | Proportion of participating PHCs without blood pressure medication stockouts at each quarter | A = Number of PHCs without blood pressure medication stockouts  B = Total number of selected PHCs  Calculation: A ÷ B | Table 3 | Supervision data |
|  | Center | Proportion of participants retained in care at participating PHCs at 6, 12, 24 months | Proportion of participants had at least one follow-up visit within 6, 12, 24 months after registration in each PHC. | Supplemental Table 4, Figure 1 | Registry data |

*Some results are reported separately in other papers from our team, including primary outcomes paper and supervision paper.

Abbreviation: BP, blood pressure; DBP, diastolic blood pressure; PHC, primary healthcare center; SBP, systolic blood pressure.

| **Supplemental Table 2.** Diversity of staff by council area at baseline, N (%). | | | | | | | |
| --- | --- | --- | --- | --- | --- | --- | --- |
|  | Total | Abaji | AMAC | Bwari | Gwagwalada | Kuje | Kwali |
|  | N=60 | N=8 | N=15 | N=8 | N=11 | N=8 | N=10 |
| Permanent |  |  |  |  |  |  |  |
| Medical Doctor | 5 (1%) | 0 (0%) | 2 (2%) | 1 (1%) | 1 (1%) | 1 (2%) | 0 (0%) |
| Paramedical Professional | 4 (1%) | 0 (0%) | 0 (0%) | 0 (0%) | 4 (4%) | 0 (0%) | 0 (0%) |
| Nurse | 70 (14%) | 7 (8%) | 21 (22%) | 12 (17%) | 11 (10%) | 4 (7%) | 15 (19%) |
| Pharmacy Staff | 5 (1%) | 0 (0%) | 1 (1%) | 3 (4%) | 1 (1%) | 0 (0%) | 0 (0%) |
| Laboratory Staff | 36 (7%) | 6 (7%) | 7 (7%) | 7 (10%) | 2 (2%) | 6 (11%) | 8 (10%) |
| CHEW | 235 (47%) | 42 (47%) | 30 (31%) | 27 (39%) | 60 (54%) | 35 (63%) | 41 (51%) |
| JCHEW | 43 (9%) | 8 (9%) | 10 (10%) | 2 (3%) | 16 (14%) | 3 (5%) | 4 (5%) |
| Health Officer | 44 (9%) | 7 (8%) | 15 (15%) | 7 (10%) | 5 (5%) | 4 (7%) | 6 (8%) |
| Other Staff ^a^ | 60 (12%) | 18 (20%) | 11 (11%) | 11 (16%) | 11 (10%) | 3 (5%) | 6 (8%) |
| **Total Permanent** | **503** | **89** | **97** | **70** | **111** | **56** | **80** |
| Per-Diem |  |  |  |  |  |  |  |
| Medical Doctor | 5 (1%) | 1 (8%) | 2 (1%) | 0 (0%) | 2 (1%) | 0 (0%) | 0 (0%) |
| Paramedical Professional | 1 (0%) | 0 (0%) | 0 (0%) | 0 (0%) | 1 (1%) | 0 (0%) | 0 (0%) |
| Nurse | 72 (12%) | 4 (33%) | 13 (6%) | 11 (12%) | 23 (14%) | 19 (31%) | 2 (3%) |
| Pharmacy Staff | 4 (1%) | 0 (0%) | 0 (0%) | 4 (4%) | 0 (0%) | 0 (0%) | 0 (0%) |
| Laboratory Staff | 66 (11%) | 3 (25%) | 24 (11%) | 18 (20%) | 9 (6%) | 5 (8%) | 7 (10%) |
| CHEW | 181 (29%) | 2 (17%) | 68 (30%) | 31 (34%) | 40 (25%) | 20 (33%) | 20 (28%) |
| JCHEW | 54 (9%) | 1 (8%) | 20 (9%) | 19 (21%) | 7 (4%) | 0 (0%) | 7 (10%) |
| Health Officer | 3 (0%) | 0 (0%) | 2 (1%) | 0 (0%) | 1 (1%) | 0 (0%) | 0 (0%) |
| Other Staff ^a^ | 236 (38%) | 1 (8%) | 94 (42%) | 8 (9%) | 80 (49%) | 17 (28%) | 36 (50%) |
| **Total Per-Diem** | **622** | **12** | **223** | **91** | **163** | **61** | **72** |
| **Total Permanent and Per-Diem** | **1,125** | **101** | **320** | **161** | **274** | **117** | **152** |
| Community Volunteers | 58 | 17 | 2 | 0 | 32 | 7 | 0 |
| **Total with Volunteers** | **1,183** | **118** | **322** | **161** | **306** | **124** | **152** |
|  |  |  |  |  |  |  |  |
|  |  |  |  |  |  |  |  |
| Permanent Healthcare Staff ^b^ | 443 | 71 | 86 | 59 | 100 | 53 | 74 |
| Per-Diem Healthcare Staff ^b^ | 386 | 11 | 129 | 83 | 83 | 44 | 36 |
| **Total Healthcare Staff** ^b^ | 829 | 82 | 215 | 142 | 183 | 97 | 110 |
| ^a^ Includes: cleaner, driver, environmental, health assistant, health attendant, health information officer, medical records, record officer, security  ^b^ Includes: Medical Doctors, Paramedical Professionals, Nurses, Pharmacy Technicians, Laboratory Technologists, CHEWs, JCHEWs, Health Officers  Abbreviations: (J)CHEW= (Junior) Community Health Extension Worker | | | | | | | |

| **Supplemental Table 3.** Sociodemographic and clinical characteristics of registered patients at baseline, by implementation period. | | | | |
| --- | --- | --- | --- | --- |
| **Characteristic, N (%)** | **Total**  **N=21,922** | **Pre-Implementation**  **N=5,506 (25.1%)** | **Implementation**  **N=16,416 (74.9%)** | **P Value** |
| Area Council |  |  |  | <.0001 |
| Abaji | 3,257 (14.9%) | 883 (16.0%) | 2,374 (14.5%) |  |
| AMAC | 7,006 (32.0%) | 1,655 (30.1%) | 5,351 (32.6%) |  |
| Bwari | 3,249 (14.8%) | 846 (15.4%) | 2,403 (14.6%) |  |
| Gwagwalada | 4,218 (19.2%) | 1,123 (20.4%) | 3,095 (18.9%) |  |
| Kuje | 1,999 (9.1%) | 369 (6.7%) | 1,630 (9.9%) |  |
| Kwali | 2,193 (10.0%) | 630 (11.4%) | 1,563 (9.5%) |  |
| Age, years, Median (IQR) | 49 (40, 58) | 48 (38, 57) | 50 (41, 58) | <.0001 |
| Female | 14,942 (68.2%) | 3,664 (66.6%) | 11,278 (68.7%) | 0.003 |
| BMI, kg/m^2^, Median (IQR) | 26.6 (23.1, 30.9) | 26.6 (23.3, 30.8) | 26.6 (23.1, 30.9) | 0.74 |
| Education |  |  |  | <.0001 |
| Never attended | 5,899 (27.0%) | 1,478 (26.9%) | 4,421 (27.1%) |  |
| Primary | 4,105 (18.8%) | 1,059 (19.3%) | 3,046 (18.7%) |  |
| Secondary | 6,123 (28.1%) | 1,596 (29.1%) | 4,527 (27.7%) |  |
| College/Post-Secondary | 4,828 (22.1%) | 1,150 (21.0%) | 3,678 (22.5%) |  |
| Professional | 751 (3.4%) | 159 (2.9%) | 592 (3.6%) |  |
| Theological | 115 (0.5%) | 46 (0.8%) | 69 (0.4%) |  |
| History of Diabetes | 974 (4.4%) | 311 (5.7%) | 663 (4.1%) | <.0001 |
| Current Smoker | 201 (0.9%) | 56 (1.0%) | 145 (0.9%) | 0.37 |
| Current Alcohol User | 774 (3.5%) | 227 (4.1%) | 547 (3.3%) | 0.006 |
| Previous Diagnosis^a^ | 10,683 (48.7%) | 2,896 (52.6%) | 7,787 (47.4%) | <.0001 |
| Among Patients with Previous Diagnosis |  |  |  |  |
| Walk-in Treated | 6,966 (65.2%) | 2,112 (72.9%) | 4,854 (62.3%) | <.0001 |
| Walk-in Controlled* | 2,059 (19.3%) | 575 (19.9%) | 1,484 (19.1%) | 0.35 |
| Among All Patients |  |  |  |  |
| Walk-in Treated* | 9,457 (43.1%) | 3,049 (55.4%) | 6,408 (39.0%) | <.0001 |
| Walk-in Controlled** | 2,792 (12.7%) | 749 (13.6%) | 2,043 (12.5%) | 0.03 |
| Systolic BP,^b^ mmHg, Median (IQR) | 154 (143, 168) | 152 (142, 164) | 154 (143, 169) | <.0001 |
| Diastolic BP,^b^ mmHg, Median (IQR) | 96 (88, 105) | 95 (88, 104) | 96 (88, 105) | <.0001 |
| Heart Rate, bpm, Median (IQR) | 82 (74, 92) | 83 (75, 92) | 82 (74, 91) | 0.01 |
| Walk-Out Treated | 20,941 (95.5%) | 5,032 (91.4%) | 15,909 (96.9%) | <.0001 |
| ^a^ Self-report or clinic knowledge of a prior diagnosis.  ^b^ Average of two measurements following a prescribed measurement protocol.  *The walk-in treatment rate can be considered the rate at the start of a patient’s baseline visit.  ** < 140/90 mmHg Following the Nigeria Hypertension Treatment Protocol  Abbreviations: AMAC, Abuja Municipal Area Council; BMI, body mass index; BP, blood pressure; HTN, hypertension; IQR, interquartile range | | | | |

**Supplemental Table 4.** Characteristics of 3-, 6-,12-, 24-month retention rate by areas.

| **Area** | **3-month retention rate, N (%)** | | | **6-month retention rate, N (%)** | | | **12-month retention rate, N (%)** | | | **24-month retention rate, N (%)** | | |
| --- | --- | --- | --- | --- | --- | --- | --- | --- | --- | --- | --- | --- |
|  | pre-implementation | implementation | P value | pre-implementation | implementation | P value | pre-implementation | implementation | P value | pre-implementation | implementation | P value |
| Abaji | 519 (58.9%) | 1469 (64.1%) | 0.914 | 628 (71.3%) | 1519 (69.4%) | 0.094 | 719 (81.6%) | 1472 (72.1%) | <0.001 | 755 (85.7%) | 1131 (78.0%) | 0.031 |
| Abuja Municipal Area Council | 639 (38.4%) | 2668 (50.7%) | 0.009 | 749 (45.0%) | 2914 (57.5%) | 0.003 | 858 (51.6%) | 2869 (61.6%) | 0.012 | 965 (58.0%) | 2128 (65.9%) | 0.029 |
| Bwari | 358 (41.9%) | 1348 (57.5%) | 0.002 | 433 (50.6%) | 1383 (62.5%) | 0.028 | 500 (58.5%) | 1356 (66.9%) | 0.649 | 535 (62.6%) | 964 (71.7%) | 0.256 |
| Gwagwalada | 729 (65.5%) | 1775 (59.0%) | 0.017 | 809 (72.7%) | 1903 (65.4%) | 0.012 | 853 (76.6%) | 1876 (68.2%) | 0.006 | 888 (79.8%) | 1476 (73.1%) | 0.096 |
| Kuje | 239 (64.8%) | 1045 (67.4%) | 0.29 | 285 (77.2%) | 1072 (72.4%) | 0.021 | 294 (79.7%) | 1006 (76.2%) | 0.113 | 302 (81.8%) | 636 (82.5%) | 0.734 |
| Kwali | 360 (56.8%) | 843 (54.4%) | 0.123 | 401 (63.2%) | 896 (59.9%) | 0.044 | 429 (67.7%) | 897 (64.3%) | 0.158 | 448 (70.7%) | 699 (71.0%) | 0.829 |
| Overall | 2844 (51.6%) | 9148 (57.2%) | 0.717 | 3305 (59.9%) | 9687 (63.1%) | 0.647 | 3653 (66.2%) | 9476 (66.8%) | 0.338 | 3893 (70.6%) | 7034 (71.8%) | 0.591 |

|  | *Baseline* | | | *Availability of free BP lowering medications (Protocol 1)* | | | | | | | *Drug revolving fund implemented* | | | | | | **Total*** |
| --- | --- | --- | --- | --- | --- | --- | --- | --- | --- | --- | --- | --- | --- | --- | --- | --- | --- |
|  | **2020** | | | **2021** | | | | **2022** | | | | | **2023** | | | |  |
| Region (n PHCs) | **Q2** | **Q3** | **Q4** | **Q1** | **Q2** | **Q3** | **Q4** | **Q1** | **Q2** | **Q3** | | **Q4** | **Q1** | **Q2** | **Q3** | **Q4** |  |
| Abaji (n=8) | 0 | 2 | 7 | 1,051ᵃ | 3,737 | 4,311 | 2,655 | 1,186 | 2,335ᵃ | 1,458 | | 1,325 | 709 | 843 | 1,086 | 1,094ᵃ | 21,799ᶜ |
| AMAC (n=15) | 163ᵃ | 656 | 103ʰ | 1,444ᵈ | 1,725 | 1,885 | 1,927 | 1,704 | 2,004 | 2,900 | | 3,294 | 1,600 | 954ᶜ | 1,223 | 1,395ᵃ | 22,977ʲ |
| Bwari (n=8) | 59 | 187 | 132 | 996 | 726 | 790 | 1,005 | 2,303 | 1,252 | 675ᵃ | | 903 | 948 | 837 | 640 | 566ᵃ | 12,019ᵇ |
| Gwagwalada (n=11) | 14 | 4 | 5ᵃ | 2,135 | 6,327 | 1,119 | 714 | 1,735 | 192ᶠ | 2,633 | | 2,828 | 1,355 | 920ᵃ | 924 | 883ᵃ | 21,788ʰ |
| Kuje (n=8) | 0 | 0 | 0 | 6 | 80 | 0 | 8,781 | 1,728ᵃ | 2,596 | 5,021 | | 1,250 | 15,345 | 13,407 | 1,295 | 338 | 49,847ᵃ |
| Kwali (n=10) | 0ᵈ | 0 | 0 | 1,121ᵇ | 6,737 | 1,062 | 2,161 | 1,820 | 1,793 | 1,305ᵃ | | 982 | 994 | 1,176 | 1,082 | 869 | 21,102ᵍ |
| **Total (n=60)** | 236ᵉ | 849 | 247ᶦ | 6,753ᵍ | 19,332 | 9,167 | 17,243 | 10,476ᵃ | 10,172ᵍ | 13,992ᵇ | | 10,582 | 20,951 | 18,137ᵈ | 6,250 | 5,145ᵈ | 149,532ᵏ |
| Primary healthcare center inventories missing: ᵃ n=1; ᵇ n=2; ᶜ n=3; ᵈ n=4; ᵉ n=5; ᶠ n=6; ᵍ n=7; ʰ n=9; ᶦ n=10; ʲ n=18; ᵏ n=40  ^†^ Blood pressure lowering medications including: amlodipine, losartan, hydrochlorothiazide, amlodipine + losartan, amlodipine + losartan +  hydrochlorothiazide | | | | | | | | | | | | | | | | | |

*P value for the trend <.0001

**Supplemental Figure 1a.** Number of 30-day doses of any BP-lowering drugs in stock by area council (Protocol 1).

|  | *Baseline* | | | *Availability of free BP lowering medications (Protocol 2)* | | | | | | | *Drug revolving fund implemented* | | | | | | **Total*** |
| --- | --- | --- | --- | --- | --- | --- | --- | --- | --- | --- | --- | --- | --- | --- | --- | --- | --- |
|  | **2020** | | | **2021** | | | | **2022** | | | | | **2023** | | | |  |
| Region (n PHCs) | **Q2** | **Q3** | **Q4** | **Q1** | **Q2** | **Q3** | **Q4** | **Q1** | **Q2** | **Q3** | | **Q4** | **Q1** | **Q2** | **Q3** | **Q4** |  |
| Abaji (n=8) | 20 | 2 | 7 | 1,051ᵃ | 3,737 | 4,311 | 2,655 | 1,186 | 2,447ᵃ | 3,514 | | 2,083 | 1,686 | 1,841 | 2,211 | 1,741ᵃ | 28,492ᶜ |
| AMAC (n=15) | 203ᵃ | 656 | 103ʰ | 1,444ᵈ | 1,740 | 1,900 | 1,963 | 1,704 | 2,004 | 3,765 | | 4,406 | 2,205 | 1,624ᶜ | 2,203 | 1,768ᵃ | 27,688ʲ |
| Bwari (n=8) | 97 | 209 | 146 | 996 | 729 | 790 | 1,005 | 2,303 | 1,252 | 989ᵃ | | 1,148 | 1,365 | 1,339 | 887 | 805ᵃ | 14,060ᵇ |
| Gwagwalada (n=11) | 15 | 4 | 5ᵃ | 2,135 | 6,327 | 1,119 | 714 | 1,735 | 192ᶠ | 3,492 | | 2,973 | 13,134 | 1,182ᵃ | 1,174 | 954ᵃ | 35,155ʰ |
| Kuje (n=8) | 0 | 0 | 0 | 6 | 80 | 0 | 8,781 | 1,728ᵃ | 2,596 | 5,696 | | 1,864 | 18,292 | 15,691 | 2,102 | 488 | 57,324ᵃ |
| Kwali (n=10) | 0ᵈ | 0 | 0 | 1,121ᵇ | 6,737 | 1,062 | 2,161 | 1,820 | 1,793 | 1,741ᵃ | | 1,240 | 1,502 | 1,712 | 1,575 | 1,401 | 23,865ᵍ |
| **Total (n=60)** | 335ᵉ | 871 | 261ᶦ | 6,753ᵍ | 19,350 | 9,182 | 17,279 | 10,476ᵃ | 10,284ᵍ | 19,197ᵇ | | 13,714 | 38,184 | 23,389ᵈ | 10,152 | 7,157ᵈ | 186,584ᵏ |
| Primary healthcare center inventories missing: ᵃ n=1; ᵇ n=2; ᶜ n=3; ᵈ n=4; ᵉ n=5; ᶠ n=6; ᵍ n=7; ʰ n=9; ᶦ n=10; ʲ n=18; ᵏ n=40  ^†^ Blood pressure lowering medications including: amlodipine, losartan, hydrochlorothiazide, amlodipine + losartan, amlodipine + losartan +  hydrochlorothiazide, amiloride + hydrochlorothiazide | | | | | | | | | | | | | | | | | |

*P value for the trend <.0001

**Supplemental Figure 1b.** Number of 30-day doses of any BP-lowering drugs in stock by area council (Protocol 2)

**
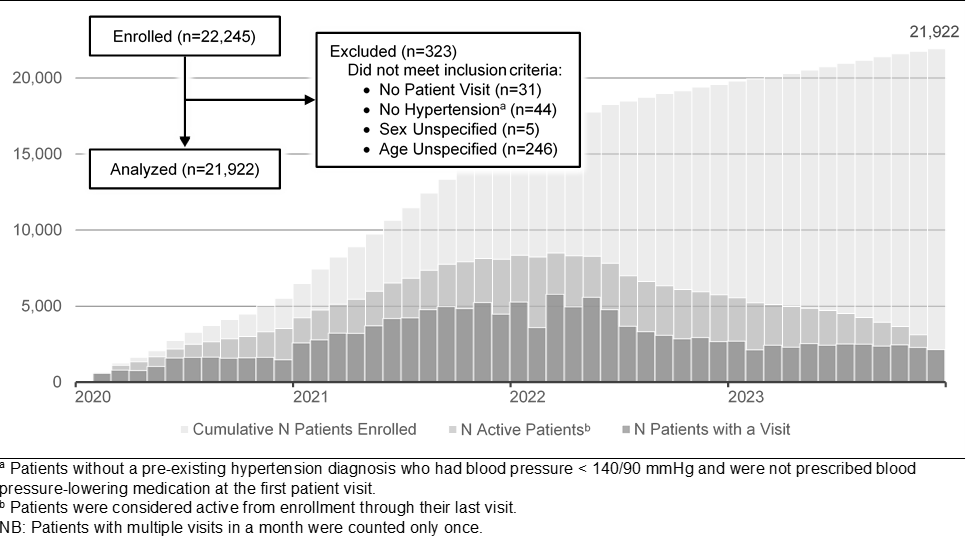
**

**Supplemental Figure 2.** Participant flowchart: enrollment and inclusion.
